# Multidimensional impact of sport types on the psychological well-being of student athletes: A multivariate investigation

**DOI:** 10.1016/j.heliyon.2024.e32331

**Published:** 2024-06-03

**Authors:** Jie Li, Zhiyu Leng, Kexin Tang, Meng Na, Yixiang Li, Syed Shah Alam

**Affiliations:** a Dept of Sport & Leisure Studies, Hoseo University, Asan, Chungcheongnam-do, South Korea; b Senior Lecturer, Institute of Disaster Prevention, Hebei Province, 065201, China; c Senior Lecturer, Xiangtan Institute of Technology, Xiangtan City, Hunan Province, China; d Graduate School of Business, Universiti Kebangsaan, Malaysia, 43600, UKM Bangi, Selangor, Malaysia; e Xiangtan Institute of Technology, Xiangtan City, Hunan Province, China; f Department of Marketing, College of Business Administration, Prince Sultan University, Riyadh, Saudi Arabia

**Keywords:** Competitive vs. non-competitive sports, Psychological resilience in athletes, Social support mechanisms, Coping strategies in sports, Cultural contexts in sports psychology, Multivariate analysis

## Abstract

The correlation between sports participation and psychological well-being is well-documented, revealing a complex interplay influenced by competition level and cultural context. This is particularly relevant in Korea, where the university sports culture significantly impacts student life. This study evaluates how competitive versus non-competitive sports affect Korean university students' psychological well-being using a quantitative approach with SmartPLS 4 for multi-group analysis. Findings reveal that competitive sports significantly enhance mental toughness and stress management through structured coping mechanisms and robust social support, improving coping strategy effectiveness by 34 % compared to non-competitive sports. Conversely, participants in non-competitive sports experience greater general well-being with a 40 % higher use of informal support. These insights suggest that university sports programs could benefit from targeted interventions incorporating specific coping strategies and social support frameworks tailored to the competitive context. This research underscores the need for precise stress management techniques and resilience-building exercises in sports curricula to optimize psychological well-being across different sports environments in Korean universities.

## 1 Introduction

In the dynamic field of sports psychology, the intricate relationship between sports participation and psychological well-being has garnered significant attention, revealing a complex interplay affecting various demographics, including university students and elite athletes. Groundbreaking studies by Purcell et al. [1] and Jovanović et al. [2] have brought to light elevated levels of psychological distress and mental health symptoms within athletic populations, signalling an urgent call for a more nuanced understanding of the myriad factors contributing to these outcomes. This burgeoning area of research suggests that while sports participation harbours the potential to enhance psychological resilience, the nature of this influence is multifaceted and subject to various mediating factors.

Conversely, insightful work by Scott et al. [3] and Foster et al. [4] has underscored the protective role that psychological well-being, such as self-esteem and mindfulness, may play in buffering athletes against the negative repercussions of competitive pressures and adverse teammate interactions. These findings highlight the beneficial aspects of sports engagement and underscore the importance of nurturing a supportive and positive sports environment. Furthermore, the research conducted by Walton et al. [5] and McLoughlin et al. [6] delves into the realm of gender differences and the cumulative impact of stress, revealing that female athletes may experience higher rates of mental health symptoms and underscoring the necessity for tailored psychological interventions.

Kuettel et al. [7] advance this discourse by categorizing distinct mental health profiles among elite athletes, illuminating this population's varied psychological experiences and support requirements. This segmentation of mental health profiles emphasizes the need for a personalized approach to psychological support in sports settings, suggesting that what benefits one athlete may not necessarily apply to another. Exploring the psychosocial dimensions of sports participation, particularly among older athletes [8], and examining the repercussions of sports-related injuries and the global COVID-19 pandemic on professional athletes' mental health [9,10], have opened new avenues for research. These studies not only corroborate the positive correlation between sports engagement and psychological well-being, such as reductions in anxiety and depression, but also highlight the critical areas necessitating further exploration.

Moreover, the literature reveals a notable gap in our understanding of the specific coping strategies athletes employ to navigate the psychological challenges of sports participation. This is particularly evident among older athletes and elite-level sports coaches [8,11], where the demands of high-performance sports can exact a significant psychological toll. Concurrently, the potential negative repercussions of sports participation, such as the emergence of aggressive and violent behavior among youth athletes [12], necessitate a balanced and comprehensive investigation into both the advantageous and detrimental outcomes of sports engagement.

Research initiatives by Jetzke et al. [13] and Arnold et al. [14] have further enriched our understanding of how the type of sport and associated extrinsic goals and organizational stressors can modulate the impact of social support on athletes' well-being. This body of work, augmented by studies from Scott et al. [3] and Cannella et al. [8], delves into the complex dynamics of social support within the competitive sports milieu, highlighting the mediating influence of psychological well-being on the nexus between teammate interactions and specific psychopathologies. The affirming impact of team sports participation on social cohesion and psychological health, as evidenced by Andersen et al. [15] and Cnen et al. [16], emphasizing the mediating role of optimism, further underscores the myriad benefits of sports engagement.

The consistently positive association between social support and psychological well-being across various studies [17,18] is mediated by a constellation of factors, including proactive coping, mindfulness, self-compassion, and the ability to savour positive experiences [19,20]. These findings resonate across different age groups and underscore the value of effective social support strategies that are attuned to the nature of the support, the relationship dynamics between the giver and receiver, and the broader cultural context [21,22].

Despite the extensive body of research illuminating the benefits and challenges associated with sports participation on psychological well-being, significant gaps persist in our comprehensive understanding of the nuanced mechanisms through which sports engagement, social support, and coping strategies collectively influence mental health outcomes. Particularly within specific cultural contexts, such as among Korean university students, the differential impacts of competitive versus non-competitive sports remain underexplored. Moreover, the potential for targeted interventions to amplify the psychological benefits of sports participation and mitigate its adverse effects is yet to be fully realized. Bridging these gaps is imperative for developing comprehensive support systems that cater to the diverse needs of athletes, fostering environments conducive to psychological resilience and well-being across various sports settings and demographic groups.

The primary objective of this study is to elucidate the impact of competitive versus non-competitive sports participation on the psychological well-being of Korean university students. Specifically, the study aims to.

1. Compare the levels of psychological well-being between students engaged in competitive sports and those participating in non-competitive sports activities.
2. Examine the role of social support in modulating the relationship between sports participation and psychological well-being.
3. Assess the mediating effects of different coping strategies on the impact of sports participation on psychological well-being.
4. Explore the potential moderating effects of the type of sport (competitive vs. non-competitive) on the interplay between social support, coping strategies, and psychological well-being.

The findings are expected to enrich the existing literature by delineating the differential effects of competitive versus non-competitive sports engagements, elucidating the roles of social support and coping strategies, and exploring their interactions within specific cultural contexts. For policymakers and educators, the insights garnered from this research will be invaluable in developing targeted interventions and support systems that are culturally sensitive and tailored to meet the diverse needs of student-athletes. These interventions can mitigate the adverse effects of sports participation, such as psychological distress and negative teammate influences, while enhancing protective factors like self-esteem, mindfulness, and resilience. Furthermore, by offering a deeper understanding of the psychosocial dynamics underpinning sports participation, the study may inform the design of university sports programs and mental health services, ultimately fostering environments that promote the holistic well-being of all students. The potential to contribute to global discussions on sports psychology and mental health through cross-cultural comparisons and the exploration of universal versus culture-specific factors further underscores the study's broader implications for enhancing psychological well-being among university students worldwide.

## 2 Psychological well-being

Psychological well-being is a multifaceted construct reflecting an individual's subjective experience and overall functioning in life. It encompasses various aspects, including emotional states, physical self-efficacy, satisfaction, and happiness, each contributing uniquely to mental health. Understanding psychological well-being involves exploring the balance between positive and negative effects, as proposed by Bradburn [23]. Fredrickson's [24] broaden-and-build theory highlights how positive emotions enhance awareness and promote exploratory thoughts and actions, while negative emotions may lead to avoidance or aggression Lazarus et al. [25]. The regulation of these emotional states plays a critical role in maintaining psychological well-being, with strategies for managing emotions proving significant [26].

Recent research has supported these concepts, showing that quality of life's psychological and physical domains significantly predict happiness and life satisfaction [27,28]. Furthermore, significant relationships have been found between well-being, leisure satisfaction, life satisfaction, and happiness, with leisure and life satisfaction serving as moderators and mediators [29,30]. The importance of health, personality traits, self-efficacy, and self-esteem in promoting psychological well-being has also been highlighted [31,32], along with the positive assessment of self-fulfillment [33].

Based on Bandura's [34] concept of self-efficacy, physical self-efficacy underscores the belief in one's ability to perform tasks requiring physical action. This belief impacts motivation, emotional responses, and performance, particularly in health behaviors, chronic disease management, and sports [35]. Satisfaction and happiness also play crucial roles in psychological well-being. Satisfaction pertains to the cognitive judgment of one's life or specific domains [36], influenced by personality and socio-economic status [37]. Happiness, or the favourable perception of one's life quality [38], is influenced by genetic set points, life circumstances, and intentional activities [39] and is associated with positive life outcomes [36].

These dimensions' dynamic and adaptable nature suggests a synergistic relationship among them [[40], [41], [42]]. Cultural variations in the concept of happiness and well-being [43,44] and determinants of subjective well-being [45,46] have been explored, along with the relationship between psychological well-being and mortality risk [47,48]. An integrative approach to well-being includes both hedonic and eudaimonic elements, encompassing emotional, psychological, and social well-being [49].

Studies over the years have underscored the interplay of emotional states, physical self-efficacy, satisfaction, and happiness in shaping psychological well-being [[50], [51], [52], [53], [54], [55]]. This comprehensive examination reveals that these dimensions are intricately connected, each pivotal role in an individual's mental health. A comparative study is especially crucial for further understanding the causal relationships and changes in psychological well-being over time, informing the development of effective interventions to enhance mental health and quality of life.

## 3 Theoretical understanding

The psychological well-being of student-athletes is a multifaceted issue influenced by the competitive or non-competitive nature of their sports participation. Self-determination theory (SDT), as expounded by Deci and Ryan [56,57], contends that well-being is bolstered when the psychological needs for competence, autonomy, and relatedness are satisfied. Athletes in competitive sports may enjoy an elevated sense of competence through formal accolades and achievements, a factor considered crucial for well-being by SDT [58]. However, these same competitive environments might impinge upon the athletes' autonomy, adversely affecting their psychological well-being [59]. In contrast, those participating in non-competitive sports might experience greater autonomy and relatedness, fostering well-being through personal development and social interactions within their sporting activities.

The relevance of SDT extends beyond sports, as it has been applied in diverse fields such as Human-Computer Interaction (HCI) [60], organizational psychology [61,62], and the study of healthy aging [63]. Research consistently shows that fulfilling basic psychological needs and autonomous motivation are linked to well-being. In contrast, the frustration of these needs and the presence of motivation are associated with ill-being [64]. The theory further emphasizes the role of intrinsic goals and moral virtues in enhancing well-being [65,66], and it suggests that workplace challenge demands can affect well-being through the satisfaction or thwarting of basic psychological needs [67]. Statistical evidence reinforces the positive correlations between motivational regulations and basic psychological needs [68].

Stress and coping, as integral aspects of the athletic experience, are conceptualized by Lazarus and Folkman's Stress and Coping Theory [25]. This theory distinguishes between problem-focused and emotion-focused coping strategies, each applicable within the sporting context. Competitive athletes often rely on problem-focused coping to tackle the direct stressors linked to performance demands [69]. In contrast, non-competitive athletes may favour emotion-focused coping to address stressors associated with personal enjoyment and sports participation [70]. Recent research, such as that by Owen et al. [71], Kalla [72], and ŞAHAN et al. [73], has reinforced the importance of coping strategies in influencing psychological well-being across various settings, including the workplace and during significant societal stressors like the COVID-19 pandemic. Cramer et al. [74] also found associations between specific coping strategies and improved mental health and subjective well-being among mental health staff.

Social Support Theory, articulated by Cobb [75] and Sarason et al. [76], posits that social relationships play a vital role in mitigating stress and improving well-being. In the demanding world of competitive sports, robust social support systems are essential in alleviating the effects of stress and contributing to athletes' psychological states [77]. Conversely, in non-competitive sports, social support is instrumental in enhancing participation enjoyment and fostering continued engagement [78]. Research spanning various demographics has consistently identified a positive correlation between social support and psychological well-being [17,18], with the nature of the support, the giver-receiver relationship, and the cultural context all playing significant roles [22].

Finally, the Competence Motivation Theory, proposed by Harter [79,80], underscores the importance of perceived competence and intrinsic motivation in activity engagement. External validation, such as awards, can strongly motivate competitive athletes, impacting their psychological well-being. For non-competitive athletes, intrinsic motivation and personal accomplishment are significant drivers of well-being [80]. Research by Fang et al. [81] and Collie [82] has emphasized the role of competence frustration and autonomous motivation in driving individuals towards success in competence-supportive activities. Studies by Wang et al. [83] and others have supported the notion that need satisfaction is linked to autonomous motivation and positive outcomes.

**Fig. 1:**
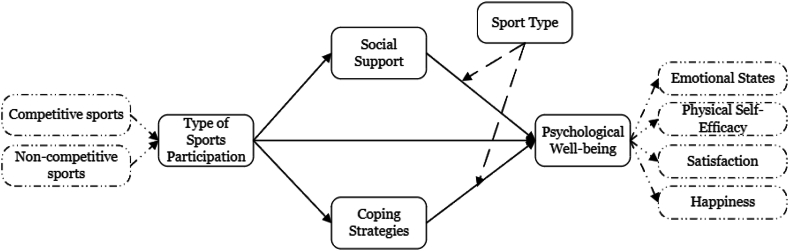
Research framework.

Collectively, these theories offer a detailed lens through which to evaluate the psychological well-being of student-athletes. They illustrate (Fig. 1) how sports participation's competitive or non-competitive nature influences the satisfaction of psychological needs, the deployment of coping strategies, and the necessity for social support. This complex approach is crucial for comprehending the complex impact of sports on student-athletes. It emphasizes the importance of considering the motivational climate and the social environment to promote athletes' well-being.

## 4 Hypothesis development

Research has consistently demonstrated that competitive athletes exhibit higher psychological well-being levels than their non-competitive counterparts [84,85]. This trend is particularly notable among athletes with a purpose-based narrative identity, showcasing elevated well-being [86]. The dynamics between mental toughness and psychological strategies are intricate, with certain strategies positively correlating with mental toughness [87]. Furthermore, an athlete's level of participation and the sport type can affect mood states and self-esteem, with the severity of post-injury mood disturbances linked to injury severity [88]. Despite the general trend of higher well-being in competitive athletes [89,90], the COVID-19 pandemic has introduced unique challenges, affecting athletes' mental health by increasing anxiety levels, albeit without diminishing resilience [91]. Elite athletes, in particular, face a heightened risk of experiencing mental health symptoms, including psychological distress [1]. Engaging in competitive sports has been shown to improve self-perceived psychophysical well-being in young individuals with physical or intellectual impairments [92]. Nevertheless, the prevalence of mental disorders among elite athletes mirrors that of the general population, underscoring the necessity for ongoing psychological support [2].

Hypothesis 1 University students in competitive sports have different psychological well-being levels than those in non-competitive sports.

The role of social support in athlete psychological health is significant, with social support and negative social interactions notably influencing psychological health across a competitive season [93]. The importance of social support in enhancing athlete satisfaction and well-being has been emphasized, with specific attention to the support from athletic trainers for injured athletes [94,95]. The positive impact of received social support on athletes' psychological well-being has been consistently highlighted [96,97], alongside the stress-buffering effects of social support, especially concerning injury-related stressors and athlete satisfaction [98]. A significant link between social support and mental health issues in college athletes has also been identified [99], reinforcing the crucial role of social support in the psychological well-being of athletes, particularly within competitive environments. The importance of social support in aiding athletes to cope with competitive demands is further supported [16,100], along with its influence on reducing psychological distress through mechanisms like self-compassion [5]. The mediation role of psychological well-being in the relationship between teammate influences and psychopathological outcomes and its contribution to school satisfaction and well-being have also been discussed [3,101]. Additionally, the positive effects of sports participation and social support on mental health and post-injury depressive and anxiety symptoms have been noted [102,103]. However, the cessation of competitive sports during the COVID-19 pandemic has been linked to poor mental health outcomes, accentuating the importance of sustained social support during such periods [91].

Hypothesis 2 Social support has a bigger impact on students' psychological well-being in competitive sports than those in non-competitive sports.

Research by Pons et al. [104] and Nicholls et al. [105] indicated that task-oriented coping strategies are positively associated with sports commitment and coping effectiveness, showcasing that athletes often employ strategies such as increased effort and planning. Contrarily, DeFreese et al. [93] discovered that social support and negative social interactions did not significantly moderate the relationships between stress and well-being. Furthermore, resilience, closely linked to coping mechanisms, has been identified to correlate with task-oriented coping and negatively with disengagement positively- and distraction-oriented coping [106]. Research by Levy et al. [107] and Johnson [108] further supports the importance of coping strategies, particularly task-oriented and problem-solving strategies, in maintaining confidence and managing long-term injuries in competitive athletes.

The potential mediating role of coping strategies in the psychological well-being of competitive athletes has been explored [[109], [110], [111]], although Daryna et al. [112] found no significant association between coping strategies and competitive anxiety. Psychological skills and mindfulness have been positively linked to well-being in student-athletes [4], and self-compassion has been associated with reduced psychological distress across various competitive levels [5]. Maladaptive perfectionism, known to induce negative emotional states in high-performance athletes, can be mitigated through strategies like cognitive restructuring and emotional calm [113], with vulnerable athletes often resorting to emotion-oriented coping strategies [114]. This underscores the intricate relationship between sports participation, coping strategies, and psychological well-being, influenced by diverse individual and situational factors.

Hypothesis 3 Coping strategies influence students' psychological well-being in competitive sports more than in non-competitive sports.

Despite findings by DeFreese et al. [93] that social support and negative social interactions did not moderate the stress-burnout or burnout-well-being relationships among American collegiate athletes, Clement et al. [95] emphasized the critical role of social support, especially from athletic trainers, in injured athletes' rehabilitation and well-being. Chatzisarantis et al. [115] and Mitchell et al. [116] both highlighted the stress-buffering effects of social support in sports, with Arnold et al. [14] examining the moderating role of social support in organizational stressors and performance, revealing that certain social support dimensions can exacerbate stress reactions. Nicholson et al. [117] and Sheridan et al. [118] discussed the relationship between sports involvement and social support, indicating the significant influence of community activities and the pivotal role of coaches, parents, and peers in youth sports experiences. Rees et al. [119] stressed the need for matching specific types of sport-relevant social support to the needs elicited by stressors in tennis.

Recent research supports the positive impact of social support on psychological well-being in sports, with a stronger effect observed in competitive settings [102,120,121]. The significance of team identification, the association between sports participation and mental health, and the influence of compassionate coaching practices are notable [103,[122], [123], [124], [125]], highlighting the role of social support in recovery from injury, its effects on well-being and performance, and its importance in sport-based programs for cancer survivors and the elderly.

Hypothesis 4 The type of sport (competitive vs. non-competitive) changes how much social support affects students' psychological well-being, with a bigger effect in competitive sports.

Sports psychology research emphasizes the crucial role of coping strategies in athletes' psychological well-being. Ntoumanis et al. [126] found that problem-focused coping is linked to positive affect, while emotion-focused coping correlates with negative affect. Secades et al. [106] highlighted that resilient athletes prefer adaptive, task-oriented coping over disengagement and distraction. The nature of stress and perceived intensity determine the selection between approach and avoidance coping [127].

DeFreese et al. [93] indicated that while athlete social support and negative social interactions affect psychological health, they may not significantly alter the stress-burnout or burnout-well-being dynamics. Gaudreau et al. [128] noted coping strategies might shift during competition, influenced by performance-goal discrepancies. Athletes' meta-experiences, or beliefs about the impact of experiences on performance, also play a role in the effectiveness of coping strategies [129].

Self-determination in sports predicts using task-oriented coping strategies, enhancing goal achievement and emotional adjustment [130]. Anshel et al. [131] observed that coping strategy use varies according to gender, skill level, and situational assessment. Mitić et al. [132] and Lee et al. [133] explored how sports performance moderates the relationship between emotional competence and coping strategies, finding certain strategies can negatively affect the relationship between negative emotions and psychological well-being in athletic coaches. Yet, Daryna et al. [112] and Kim et al. [101] reported no significant link between coping strategies and competitive anxiety or the impact of spectator behaviour.

Rodríguez-Bravo et al. [134] and Litwic-Kaminska [135] stressed the positive effects of physical-sport activities on well-being, focusing on the role of positive appraisals. Hagan Jr [136]. associated negative religious coping with unpleasant emotions in elite student-athletes, illustrating the complex relationship between coping strategies and psychological well-being, influenced by sports performance, specific strategies, and personal appraisals.

Hypothesis 5 The type of sport (competitive vs. non-competitive) influences the effect of coping strategies on students' psychological well-being, with a more significant impact observed in competitive sports.

## 5 Research methodology

### 5.1 Target population and sampling

This quantitative study is designed to delve into the psychological well-being of Korean university students, with a nuanced examination of the variances between those participating in competitive versus non-competitive sports. Aiming to quantitatively assess the influence of social support and coping strategies on students' well-being, this research endeavours to illuminate the intricate dynamics present within the sports participation context at Korean universities. The survey was administered online to enhance participation convenience and expand the study's reach. The study zeroes in on the university student demographic within Hunan Province, utilizing a cluster random sampling technique for participant selection. The province is segmented into five geographic zones: North, South, East, West, and Central. One university from each zone is chosen for inclusion: Shonan University, Hunan University of Technology, Jishou University, Hunan University, and Huaihua University. To distribute 200 questionnaires per university, the goal was to amass 1000 questionnaires across all chosen sites. Ultimately, the study garnered 975 valid responses, following the exclusion of incomplete submissions. A preliminary pilot test involving 50 questionnaires was conducted to evaluate the survey's clarity, relevance, and effectiveness. Insights gained from this pilot phase informed subsequent refinements to the questionnaire, optimizing it for the main study population.

### 5.2 Participants

The questionnaire is structured to collect basic demographic information detailed in Table 1, facilitating a diverse and comprehensive sample analysis. The age distribution of respondents predominantly falls within the 18–21 range, accounting for over half of the sample, followed closely by the 22–25 age group. This skew towards younger adults is particularly significant, reflecting the study's focus on a demographic navigating critical developmental milestones.

**Table 1:** Demographics distribution.

Demographic Characteristics	Total Respondents (N = 975)	Percentage (%)
Age		
18-21	525	53.8
22-25	400	41.0
26-29	45	4.6
30+	5	0.5
**Gender**		
Male	500	51.3
Female	475	48.7
**Year of Study**		
First Year	240	24.6
Second Year	235	24.1
Third Year	250	25.6
Fourth Year	250	25.6
**Major/Field of Study**		
STEM (Science, Tech, Engineering, Math)	350	35.9
Humanities and Social Sciences	225	23.1
Business and Economics	250	25.6
Arts and Physical Education	150	15.4
**Type of Sports Participation**		
Competitive	325	33.3
Non-Competitive	650	66.7
**Hours per Week Dedicated to Sports**		
0–2 h	375	38.5
3–5 h	450	46.2
6–10 h	125	12.8
11+ hours	25	2.6
**Participation in Sports Teams or Clubs**		
Yes	500	51.3
No	475	48.7

Gender representation within the study is nearly balanced, with a slight majority of male participants. This equitable distribution allows exploring gender-specific nuances in sports participation and its impact on well-being. Research indicates that gender can influence the choice of sporting activities and the psychological outcomes associated with sports participation [137], making this aspect of the demographic breakdown crucial for a nuanced analysis.

The study's participants span all four years of university study, offering a unique opportunity to examine how sports participation impacts psychological well-being across different stages of the university experience. This longitudinal perspective can reveal how engagement in sports interacts with academic pressures and social changes, potentially influencing students' mental health over time [83].

Diverse academic disciplines, from STEM to Arts and Physical Education, are represented among the respondents, reflecting the varied interests of university students. Previous research suggests that students' academic majors can significantly influence their extracurricular activities, including sports, potentially affecting their social networks and stress levels [138].

A striking find from the study is the predominance of non-competitive sports participation, with two-thirds of the sample engaging in recreational rather than competitive sports. While competitive sports are often associated with higher stress levels due to performance pressures, non-competitive sports may offer more recreational benefits without the accompanying stress, potentially leading to different outcomes in terms of psychological well-being [78].

Furthermore, the distribution of hours dedicated to sports per week suggests a moderate engagement in physical activities, with a significant portion of students spending 0–5 h in sports. This moderate engagement is intriguing, as it may represent a balanced approach to sports participation, possibly mitigating the risk of burnout while offering the psychological benefits of physical activity [139].

The near-even split between students participating in sports teams or clubs and those not participating provides a fertile ground for examining the social aspects of sports engagement. Participation in sports teams can offer a sense of belonging and support, pivotal for mental health. However, it also comes with its own set of challenges, including the potential for increased stress and conflict [140].

### 5.3 Research instrument development

The constructs of interest, including Types of Sports Participation, Psychological Well-Being (comprising Emotional States, Happiness, Physical self-efficacy, and Satisfaction) [141,142], Coping Strategy, and Social Support, are pivotal to exploring the dynamics within this context (refer to Table 2).

**Table 2:** Indicator loading and composite reliability of variables and indicators.

Construct	Code	Items	Competitive Sports					Non-Competitive Sports				
			OL	VIF	CA	CR	AVE	OL	VIF	CA	CR	AVE
COP	COP1	When faced with sports-related stress, I focus on what I can do to solve the problem.	0.808	1.656	0.857	0.914	0.780	0.837	1.785	0.855	0.912	0.775
	COP2	I use relaxation techniques (e.g., deep breathing and meditation) to cope with stress in sports.	0.927	3.264				0.901	2.461			
	COP3	I seek advice or support from others when I encounter difficulties in my sports activities.	0.910	3.002				0.902	2.513			
Cs	CS1	Rate the level of competition in your primary sport (e.g., local, regional, national, international).	0.946	4.428	0.877	0.925	0.805	0.924	3.106	0.837	0.903	0.757
	CS2	How often do you participate in competitive events or matches?	0.920	3.771				0.899	2.760			
	CS3	Do you engage in systematic training with the goal of improving competitive performance?	0.821	1.802				0.779	1.533			
ES	ES1	I generally feel positive about my life.	0.802	1.777	0.846	0.897	0.685	0.847	2.042	0.843	0.895	0.682
	ES2	I feel emotionally balanced and stable.	0.868	2.233				0.879	2.643			
	ES3	I often experience feelings of joy and contentment.	0.809	1.785				0.722	1.464			
	ES4	I feel in control of my emotions, even in challenging situations.	0.830	1.942				0.847	2.151			
HAP	HAP1	Participating in sports activities makes me happy.	0.930	3.269	0.920	0.950	0.862	0.912	2.690	0.900	0.938	0.834
	HAP2	I feel a sense of joy and excitement during or after sports activities.	0.924	3.318				0.898	2.666			
	HAP3	My sports experiences contribute positively to my overall happiness.	0.931	3.529				0.929	3.396			
NCS	NCS1	Do you participate in sports activities more for enjoyment than competition?	0.865	2.388	0.881	0.918	0.737	0.887	2.998	0.883	0.920	0.742
	NCS2	How often do you engage in sports activities without keeping score or competing against others?	0.852	2.227				0.805	1.853			
	NCS3	Do your sports activities involve casual group participation or individual practice without a competitive goal?	0.842	2.181				0.836	2.034			
	NCS4	Do you engage in sports activities without a formal coach or training regimen?	0.875	2.508				0.913	3.486			
PSE	PSE1	I feel confident in my ability to meet the physical demands of my sport.	0.914	2.713	0.880	0.926	0.807	0.912	2.766	0.880	0.926	0.807
	PSE2	I believe I can improve my sports performance through practice and effort.	0.896	2.525				0.910	2.743			
	PSE3	I am capable of learning new and challenging physical skills.	0.884	2.221				0.872	2.103			
SAT	SAT1	I am satisfied with my current level of sports performance.	0.867	2.570	0.917	0.942	0.802	0.836	2.109	0.898	0.929	0.766
	SAT2	My participation in sports brings me a sense of fulfillment.	0.909	3.398				0.900	3.111			
	SAT3	I feel that my sports-related goals and desires are being met.	0.905	3.358				0.917	3.485			
	SAT4	I am content with balancing my sports activities and other life areas.	0.899	3.023				0.845	2.194			
SS	SS1	I feel supported by my teammates/coach/family in my sports endeavors.	0.928	4.211	0.948	0.962	0.865	0.909	3.322	0.922	0.945	0.811
	SS2	My social network understands and respects the time I dedicate to sports.	0.940	4.898				0.908	3.300			
	SS3	I have someone in my sports circle I can talk to about problems related to sports.	0.937	4.622				0.924	3.838			
	SS4	When I am feeling down about my sports performance, I have people who uplift me.	0.915	3.601				0.861	2.413			
ST	ST1	My primary sport is team-based (e.g., football, basketball)/individual-based (e.g., tennis, swimming).	0.920	3.725	0.930	0.950	0.826	0.891	2.721	0.905	0.933	0.778
	ST2	The nature of my primary sport is endurance-based (e.g., marathon, cycling)/strength-based (e.g., weightlifting).	0.912	3.622				0.859	2.341			
	ST3	My primary sport requires high physical contact (e.g., rugby, martial arts)/minimal physical contact (e.g., badminton, golf).	0.900	3.218				0.866	2.621			
	ST4	The environment of my primary sport is outdoor (e.g., soccer, rowing)/indoor (e.g., volleyball, gymnastics).	0.902	3.267				0.911	3.346			

COP→ Coping Strategy, PWB-→ Psychological Well-Being, SS→ Social Support, TSP→ Types of Sports Participation, Cs→ Competitive Sprots, ES→ Emotional States, HAP→ Happiness, NCS→ Non-Competitive Sports, PSE→ Physical Self Efficacy, SAT→ Satisfaction, ST→ Sports Type.

For non-competitive sports participants, the high discriminant validity of constructs related to Non-Competitive Sports (NCS) and Happiness (HAP) underscores the enjoyment and intrinsic satisfaction derived from sports participation.

Competitive Sports Participation is gauged through items assessing the level of competition, frequency of competitive events, and engagement in systematic training to enhance performance. These items are inspired by seminal works in the field [70,78,143], reflecting the intensity and dedication associated with competitive sports. Conversely, Non-Competitive Sports Participation is measured through items that explore participation motivated more by enjoyment than competition, engagement in sports activities without keeping score, and involvement in casual group participation or individual practice without a competitive goal, drawing on the conceptual frameworks provided by scholars like Smith et al. [144], Coakley [145] and Eime et al. [139].

Psychological Well-Being, a multifaceted construct, encompasses Emotional States, Happiness, Physical Self Efficacy, and Satisfaction. The development of Emotional States and Happiness items, particularly within the sports context, is informed by Ryff's Scales of Psychological Well-Being [49] and research on sports and happiness [146]. Items measuring Physical Self-efficacy are constructed based on Bandura's self-efficacy theory, tailored to the physical domain [147]. At the same time, those assessing Satisfaction with sports participation reflect constructs from sports psychology literature [148].

Coping Strategy, crucial for managing sports-related stress, is measured through items inspired by Lazarus and Folkman's stress and coping paradigm [25], adapted for sports-specific contexts. Similarly, Social Support, vital for psychological well-being, is gauged using items based on Cutrona and Russell's social support typologies [149], modified for relevance to sports environments. Lastly, the Sports Type category integrates aspects from sports classification literature, including items developed to categorize sports by team dynamics, physical demand, contact level, and environment [150].

The questionnaire was subjected to a rigorous back-translation process to cater specifically to Korean students' linguistic understanding. Initially crafted in English, it was translated into Korean and then back into English by an independent professional translator, ensuring the final Korean version remained true to the original content while being fully comprehensible to Korean participants.

### 5.4 Technique for data analysis

In this study, we employed SmartPLS 4 for meticulous data analysis to ensure the robustness and reliability of psychological measures across various sports participation groups, including a detailed assessment of the higher order construct of Psychological Well-Being, which encompasses Emotional States, Happiness, Physical Self-Efficacy, and Satisfaction. Reliability of these measures was thoroughly evaluated using Composite Reliability (CR), Average Variance Extracted (AVE), and Cronbach's Alpha, with high values confirming strong and consistent internal consistency across participants. The validity of the constructs, including the multifaceted Psychological Well-Being, was carefully verified through an examination of outer loadings which were significantly higher on their intended factors compared to cross-loadings, thus ensuring clear discriminability of the various dimensions assessed. Additionally, we checked for multicollinearity among variables using the Variance Inflation Factor (VIF), with all values reported well within acceptable limits. Our structural model analysis particularly highlighted critical relationships, which appeared notably stronger in competitive sports settings, indicating higher coping demands due to competitive stress. Furthermore, a Multigroup Analysis (MICOM) was conducted to ensure measurement invariance across different groups, thereby validating our capability to make reliable comparisons and apply our findings universally across varied sporting contexts. This comprehensive analysis framework supports the integrity and applicability of our research, providing a robust basis for subsequent interventions aimed at enhancing mental health and well-being among university athletes.

### 5.5 Ethical consideration

This study prioritizes the privacy and confidentiality of all participants in strict adherence to the ethical standards outlined in the Helsinki Declaration. Sensitive personal data is not solicited, and participants are thoroughly briefed on the study's goals. Informed consent is obtained from each participant before survey commencement, ensuring voluntary participation and the ethical integrity of the research process.

## 6 Data analysis

### 6.1 Reliability statistics of the constructs

**Fig. 2:**
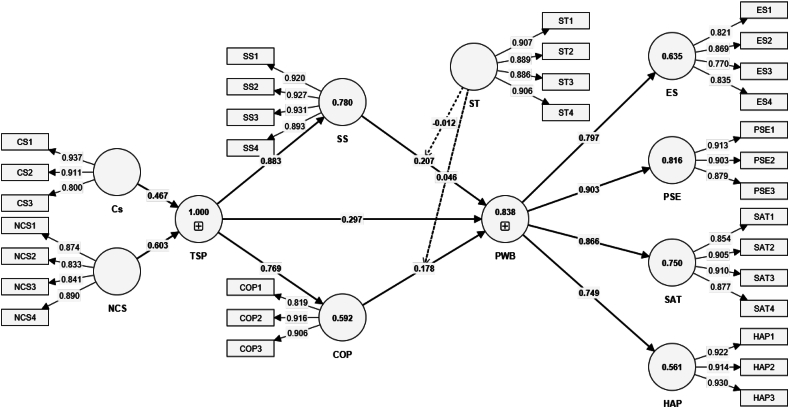
Measurement Model **Abbreviations:** COP→ coping strategy, PWB-→ psychological well-being, SS→ social support, TSP→ types of sports participation, Cs→ competitive sports, ES→ emotional states, HAP→ happiness, NCS→ non-competitive sports, PSE→ physical self-efficacy, SAT→ satisfaction, ST→ sports type.

Table 2 outlines the indicator loading and Cronbach's Alpha of variables and indicators, comparing constructs between competitive and non-competitive sports participants among Korean university students (refer to Fig. 2). Coping Strategy (COP) and Social Support (SS) show strong indicator loadings and composite reliability across competitive and non-competitive sports participants. This suggests that irrespective of the sports type, coping strategies and social support play a significant role in students' sports experiences. The high-reliability scores (CR above 0.9 and AVE above 0.7 for most constructs) indicate that the constructs are measured consistently within the sample, providing a reliable basis for analysis. Types of Sports Participation (TSP), differentiated into competitive sports (Cs) and non-competitive sports (NCS), reveal distinct patterns in how sports types influence psychological constructs. Psychological Well-Being (PWB), encompassing Emotional States (ES), Happiness (HAP), Physical Self Efficacy (PSE), and Satisfaction (SAT), demonstrates strong indicator loadings across both groups but with notable distinctions. All the construct reliability denoted checked with Cronbach's Alpha was also above the threshold of 0.7 signifying high reliability.

### 6.2 Discriminant validity

As shown in Table 3, Discriminant validity assesses each construct's distinctiveness within the competitive and non-competitive sports contexts. Notably, constructs such as Coping Strategy (COP), Competitive Sports (Cs), Emotional States (ES), and Social Support (SS) demonstrate high discriminant validity, indicated by their higher loadings on their respective factors compared to cross-loadings with other constructs. This suggests that these constructs are well-defined and distinct from one another within both groups, aligning with Fornell and Larcker's (1981) criteria for assessing discriminant validity. In competitive sports, constructs like Physical self-efficacy (PSE) and Satisfaction (SAT) show strong associations with their respective items, reflecting the intrinsic motivation and goal achievement that competitive environments foster.

**Table 3:** Discriminant validity.

	Competitive Sports											Non-Competitive Sports										
	COP	Cs	ES	HAP	NCS	PSE	SAT	SS	ST	ST x COP	ST x SS	COP	Cs	ES	HAP	NCS	PSE	SAT	SS	ST	ST x COP	ST x SS
COP																						
Cs	0.834											0.772										
ES	0.773	0.834										0.572	0.842									
HAP	0.692	0.653	0.581									0.510	0.600	0.412								
NCS	0.656	0.802	0.835	0.616								0.782	0.816	0.736	0.475							
PSE	0.502	0.849	0.791	0.708	0.872							0.813	0.832	0.729	0.654	0.844						
SAT	0.787	0.787	0.691	0.584	0.812	0.834						0.692	0.674	0.552	0.523	0.831	0.728					
SS	0.830	0.819	0.804	0.669	0.847	0.815	0.795					0.791	0.845	0.781	0.475	0.827	0.848	0.717				
ST	0.834	0.809	0.752	0.681	0.847	0.834	0.862	0.875				0.812	0.788	0.665	0.554	0.875	0.847	0.752	0.808			
ST x COP	0.493	0.398	0.305	0.253	0.464	0.464	0.448	0.442	0.468			0.506	0.327	0.190	0.090	0.338	0.444	0.212	0.309	0.426		
ST x SS	0.505	0.448	0.336	0.224	0.465	0.496	0.474	0.461	0.481	0.819		0.372	0.355	0.175	0.155	0.414	0.447	0.279	0.371	0.453	0.846	

COP→ Coping Strategy, PWB-→ Psychological Well-Being, SS→ Social Support, TSP→ Types of Sports Participation, Cs→ Competitive Sprots, ES→ Emotional States, HAP→ Happiness, NCS→ Non-Competitive Sports, PSE→ Physical Self Efficacy, SAT→ Satisfaction, ST→ Sports Type.

### 6.3 Inter-composite covariances

Table 4 presented in the inter-composite covariances, illustrating the relationships between constructs across competitive and non-competitive sports participants. The high covariance between Coping Strategy (COP) and Social Support (SS) in both groups highlights the interplay between individual coping mechanisms and the perceived support from one's social network in managing sports-related stress. This finding aligns with Lazarus and Folkman's [25] transactional model of stress and coping, emphasizing the role of social support as a buffer against stress. Moreover, the covariance between Emotional States (ES), Happiness (HAP), and Satisfaction (SAT) with Physical Self Efficacy (PSE) in competitive sports participants indicates a strong relationship between physical confidence, emotional well-being, and satisfaction with sports participation. This suggests that for competitive athletes, believing in one's physical capabilities is closely linked to positive emotional states and overall satisfaction with sports involvement.

**Table 4:** Inter-composite covariances.

	Competitive Sports										Non-Competitive Sports									
	COP	Cs	ES	HAP	NCS	PSE	PWB	SAT	SS	ST	COP	Cs	ES	HAP	NCS	PSE	PWB	SAT	SS	ST
COP	0.883										0.881									
Cs	0.730	0.897									0.660	0.870								
ES	0.660	0.717	0.828								0.495	0.707	0.826							
HAP	0.610	0.594	0.516	0.929							0.448	0.534	0.361	0.913						
NCS	0.774	0.768	0.719	0.556	0.858						0.681	0.705	0.642	0.423	0.861					
PSE	0.827	0.768	0.684	0.639	0.771	0.898					0.749	0.719	0.634	0.584	0.743	0.898				
SAT	0.701	0.710	0.608	0.539	0.731	0.771	0.881	0.895			0.608	0.590	0.485	0.473	0.742	0.649	0.835	0.875		
SS	0.791	0.843	0.768	0.626	0.821	0.838	0.877	0.741	0.930		0.707	0.791	0.699	0.438	0.836	0.764	0.801	0.653	0.901	
ST	0.749	0.793	0.667	0.631	0.796	0.848	0.872	0.798	0.824	0.909	0.712	0.689	0.587	0.503	0.783	0.845	0.819	0.680	0.739	0.882

COP→ Coping Strategy, PWB-→ Psychological Well-Being, SS→ Social Support, TSP→ Types of Sports Participation, Cs→ Competitive Sprots, ES→ Emotional States, HAP→ Happiness, NCS→ Non-Competitive Sports, PSE→ Physical Self Efficacy, SAT→ Satisfaction, ST→ Sports Type.

For non-competitive sports participants, the covariance between Non-Competitive Sports (NCS) and Happiness (HAP) suggests that engagement in sports for enjoyment significantly correlates with happiness. This highlights the importance of leisurely sports participation as a key contributor to psychological well-being, supporting findings by Eime et al. [139] that emphasize the mental health benefits of non-competitive sports participation.

### 6.4 Structural model analysis

**Fig. 3:**
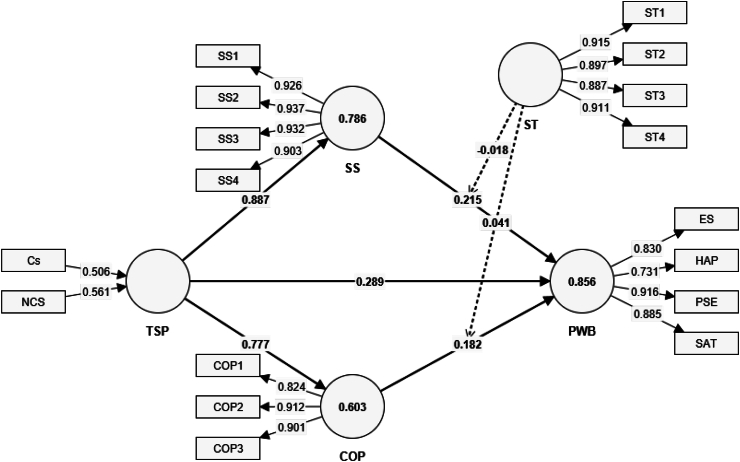
Structural model.

The exploration of how sports participation impacts the psychological well-being of Korean university students reveals intricate dynamics between the types of sports engagement, coping strategies employed, the social support received, and their collective influence on mental health. This study, through its structural model analysis (refer to Table 5, see Fig. 3) offers a profound understanding of these relationships, differentiating between competitive and non-competitive sports contexts to shed light on nuanced pathways that lead to enhanced well-being among university athletes.

**Table 5:** Results of the structural model.

Hypothesis Path	Competitive Sports		Non-Competitive Sports	
	T statistics	P values	T statistics	P values
TSP - > COP	39.117	0.000	24.499	0.000
SS - > PWB	8.104	0.000	2.142	0.032
COP - > PWB	6.564	0.000	3.773	0.000
TSP - > SS - > PWB	8.188	0.000	2.138	0.033
TSP - > COP - > PWB	6.471	0.000	3.882	0.000

COP→ Coping Strategy, PWB-→ Psychological Well-Being, SS→ Social Support, TSP→ Types of Sports Participation, Cs→ Competitive Sprots.

Significant findings emerge from the analysis, indicating that the type of sports participation directly influences students' coping strategies when faced with sports-related stress. With T statistics reaching 39.117 in competitive sports and 24.499 in non-competitive contexts and P values standing firmly at 0.000 for both, the evidence suggests a robust impact of sports participation on coping mechanisms. This effect is notably more pronounced in competitive sports, where the pressures and challenges are inherently higher, necessitating more sophisticated coping strategies. This observation aligns with the broader literature, Nicholls et al. [69], who emphasize the critical role of coping in managing the psychological demands of sports.

Further analysis of the pathways from social support and coping strategies to psychological well-being uncovers significant relationships across competitive and non-competitive sports. The pathways exhibit higher T statistics for competitive sports, underscoring the vital role of social support and effective coping in fostering mental health. These findings resonate with the buffering hypothesis proposed by Cohen et al. [151], which suggests that social support can mitigate the adverse effects of stress, and the transactional theory of stress and coping by Lazarus and Folkman [25], emphasizing the dynamic interplay between individuals and their environments in managing stress.

Moreover, the mediated pathways—where social support and coping strategies act as intermediaries between sports participation and well-being—highlight the complex interconnections within this ecosystem. Significant effects in these pathways, across both types of sports participation, suggest that the journey from sports engagement to psychological well-being traverses through the supportive and coping resources available to the athlete. This intricate dance between the variables underpins the ecological systems theory [152], which posits that multiple layers of environmental systems influence individual development. The stronger effects observed in competitive sports might reflect the heightened stress levels in such environments, pointing to the need for robust support and coping strategies. On the other hand, the significant impacts in non-competitive contexts suggest that recreational sports bolster psychological well-being through enjoyment and leisure, contributing positively to students' mental health landscapes.

### 6.5 Multigroup analysis

The Multigroup Analysis (MICOM) and subsequent tests performed in this study offer valuable insights into the compositional invariance and equality of variances across different constructs related to sports participation and its effects on psychological well-being. Table 6, Table 7, Table 8 show the nuanced differences and similarities in how competitive and non-competitive sports participation influences coping strategies, social support, and overall psychological well-being.

**Table 6:** MICOM: Compositional invariance.

	Original correlation	Correlation permutation mean	5.0 %	Permutation p-value
COP	1.000	1.000	0.999	0.266
PWB	1.000	1.000	0.999	0.189
SS	1.000	1.000	1.000	0.036
TSP	0.999	0.999	0.996	0.466

COP→ Coping Strategy, PWB-→ Psychological Well-Being, SS→ Social Support, TSP→ Types of Sports Participation, Cs→ Competitive Sprots.

**Table 7:** MICOM: Equal variances.

	Original difference	Permutation mean difference	2.5 %	97.5 %	Permutation p-value
COP	−0.104	0.001	−0.124	0.125	0.100
PWB	−0.105	−0.001	−0.125	0.124	0.104
SS	−0.094	−0.001	−0.124	0.125	0.144
TSP	−0.102	0.000	−0.126	0.125	0.114

COP→ Coping Strategy, PWB-→ Psychological Well-Being, SS→ Social Support, TSP→ Types of Sports Participation, Cs→ Competitive Sprots.

**Table 8:** Multigroup analysis test results.

Hypothesis Path		Difference (Competitive Sports - Non-Competitive Sports)	1-tailed (Competitive Sports vs Non-Competitive Sports) p-value	2-tailed (Competitive Sports vs Non-Competitive Sports) p-value	Support
H1	TSP - > COP	0.082	0.013	0.026	Yes
H2	SS - > PWB	0.191	0.001	0.002	Yes
H3	COP - > PWB	0.104	0.020	0.040	Yes
H4	TSP - > SS - > PWB	0.170	0.001	0.001	Yes
H5	TSP - > COP - > PWB	0.094	0.007	0.013	Yes

COP→ Coping Strategy, PWB-→ Psychological Well-Being, SS→ Social Support, TSP→ Types of Sports Participation, Cs→ Competitive Sprots.

Compositional invariance tests, as shown in Table 6, confirm the consistency of construct measurements across groups, with original correlations and correlation permutation means achieving perfect or near-perfect scores (1.000 and 0.999). This high level of invariance, especially with permutation p-values (ranging from 0.036 to 0.466), suggests that the constructs of Coping Strategy (COP), Psychological Well-Being (PWB), Social Support (SS), and Types of Sports Participation (TSP) are measured similarly across competitive and non-competitive sports participants.

The analysis of equal variances reveals slight differences between groups, as indicated by the original difference and permutation mean difference. However, the permutation p-values (ranging from 0.100 to 0.144) suggest that these differences are not statistically significant. This implies that the variance within each construct—COP, PWB, SS, and TSP—is relatively consistent across competitive and non-competitive sports participants, allowing for direct comparisons and hypothesis testing without concern for substantial disparities in variance that could skew results.

The multigroup analysis test results clearly show how competitive and non-competitive sports participation distinctly impacts coping strategies, social support, and psychological well-being. Notably, the paths from TSP to COP, SS to PWB, and mediated paths (TSP - > SS - > PWB and TSP- > COP- > PWB) show statistically significant differences, suggesting that the type of sports participation does influence how coping strategies and social support contribute to psychological well-being.

Importantly, the findings affirm that the direct and mediated influences of sports participation type on psychological well-being are significant, reinforcing the need for tailored approaches to support athletes' mental health across different sporting environments. This nuanced understanding can guide educators, coaches, and policymakers in developing more effective support mechanisms, enhancing university students' psychological well-being in both competitive and non-competitive sports.

## 7 Discussion on the findings

Through this empirical lens, the study navigates the complex interrelations between competitive and non-competitive sports and their respective impacts on students' mental health, drawing from a breadth of research that ranges from the benefits of competitive sports on psychological well-being [84,85] to the nuanced challenges introduced by the COVID-19 pandemic [91].

The multigroup analysis reveals that the type of sports participation (TSP) significantly affects coping strategies (COP), with competitive sports showing a more substantial impact (T statistics: 39.117 for competitive sports and 24.499 for non-competitive sports; P values: 0.000 for both). This aligns with research suggesting that competitive athletes often develop more sophisticated coping mechanisms due to their higher pressures [84,85]. The hypothesis test results further support this, showing a difference in the influence of TSP on COPbetween competitive and non-competitive sports (Difference: 0.082; 1-tailed P-value: 0.013; 2-tailed P-value: 0.026), indicating that competitive athletes are more likely to engage in effective coping strategies, corroborating the findings of Houltberg et al. [86] and Crust et al. [87].

Social support's impact on psychological well-being (SS - > PWB) is also significant across both groups but exhibits a stronger effect in competitive sports (T statistics: 8.104 for competitive sports vs. 2.142 for non-competitive sports; P values: 0.000 and 0.032, respectively). This is consistent with literature highlighting the crucial role of social support in athlete satisfaction and well-being [93,94]. The hypothesis test results underscore the differential impact, showing a more pronounced effect in competitive sports (Difference: 0.191; 1-tailed P-value: 0.001; 2-tailed P-value: 0.002), suggesting that the competitive environment amplifies the need for and benefits of social support.

Furthermore, the analysis of coping strategies' influence on psychological well-being (COP - > PWB) reveals significant pathways in both competitive and non-competitive contexts, with a notably higher impact in competitive sports (T statistics: 6.564 for competitive sports vs. 3.773 for non-competitive sports; P values: 0.000 for both). This finding supports that coping strategies are vital in managing sports-related stress and enhancing mental health, especially in competitive settings [104,105].

The mediated pathways (TSP - > SS - > PWB and TSP - > COP - > PWB) further highlight the intricate relationships between types of sports participation, social support, coping strategies, and psychological well-being. The significant differences observed in these pathways, particularly pronounced in competitive sports (Differences: 0.170 and 0.094 for the respective paths; 1-tailed P values: 0.001 and 0.007; 2-tailed P values: 0.001 and 0.013), illustrate the complex mechanisms through which sports participation impacts mental health. These results echo the literature on the mediating role of social support and coping strategies in enhancing athletes' psychological well-being [16,100].

The multigroup analysis sheds light on the differential impacts of competitive and non-competitive sports on psychological well-being, mediated by coping strategies and social support. These findings not only validate the hypotheses developed from the literature but also offer practical insights for designing targeted interventions that cater to the unique needs of athletes across different sports contexts.

## 8 Implications of this study

### 8.1 Theoretical implications

This research into the psychological well-being of university students engaged in sports, distinguishing between competitive and non-competitive participation, offers significant theoretical contributions to our comprehension of human motivation, stress management, and social networking's role. Central to this study's theoretical implications is the Self-Determination Theory (SDT), which argues that fulfilling autonomy, competence, and relatedness needs is crucial for well-being. The differences observed between competitive and non-competitive athletes underscore the complex interaction of these needs within the sports context, suggesting unique manifestations of SDT's principles across sports types and providing insights for optimizing athlete engagement and satisfaction.

Additionally, the findings on coping strategies revitalize Lazarus and Folkman's Stress and Coping Theory, especially in the context of competitive sports pressures. This underscores the theory's assertion of stress management as a dynamic process and calls for further investigation into specific coping mechanisms tailored to competitive sports challenges. Moreover, the Social Support Theory's stress-buffering effects claim is substantiated by the greater impact of social support on competitive athletes' well-being. This reaffirms the theory and suggests a reevaluation of social support dynamics within athletic environments, highlighting the need for customized support systems.

Furthermore, the Competence Motivation Theory, which associates feelings of competence and achievement with motivation and well-being, is particularly relevant for understanding competitive sports' psychological outcomes. This study suggests examining how competence motivation functions across various sports participation types, emphasizing the theory's adaptability.

This study validates and extends the scope of essential psychological theories in sports psychology, illuminating the complex mechanisms that underlie athlete motivation, stress management, and social interactions. These theoretical insights lay the foundation for future research and practical interventions to improve athletes' mental health and motivational climates across sports participation spectrums.

### 8.2 Practical implications

This study's insights uncover how different types of sports participation influence coping strategies, social support, and overall mental health, offering a detailed guide for enhancing university athletes' support systems.

Educators and coaches can use these findings to create environments that foster competitive excellence and the intrinsic enjoyment of sports participation. The study highlights the importance of incorporating mental toughness and resilience exercises into sports curriculums, focusing on developing effective stress management techniques for the competitive landscape. This approach aids athletes in managing competition pressures and fostering essential psychological well-being components, such as competence and autonomy.

The significant role of social support in boosting psychological health, particularly for competitive athletes, emphasizes the need to build strong support networks within sports teams. Initiatives like team-building activities and mentorship programs can cultivate a culture of mutual support and collective growth, enhancing athletes' psychological safety and resilience.

Sports psychologists can greatly benefit from these findings by designing targeted interventions that address athletes' unique challenges. Tailored coping skills training, including mindfulness, visualization, and emotional regulation, equips athletes with stress management tools. Additionally, advocating for psychological support services' integration within sports organizations ensures athletes' access to essential mental health resources.

Policymakers and sports program designers are encouraged to develop policies and programs reflecting student-athletes diverse needs. Promoting a balanced approach to sports participation, valuing personal growth and community alongside competitive success, can create a more inclusive and psychologically supportive sports culture. Implementing guidelines ensuring all athletes access mental health resources, regardless of competition level, is vital.

Ultimately, this study advocates for a holistic approach to university-level sports participation, acknowledging the intricate interplay between physical activity, psychological well-being, and social support. By prioritizing the development of effective coping mechanisms, fostering strong social networks, and creating inclusive sports programs, educators, coaches, psychologists, and policymakers can support student-athletes holistic development. These initiatives improve athletes' immediate well-being and provide skills and experiences that contribute to their long-term health and happiness, highlighting sports' critical role in promoting comprehensive personal development.

## 9 Conclusion and future recommendations

The comprehensive analysis of the psychological well-being of university students engaged in competitive and non-competitive sports has unearthed critical insights into the dynamics of sports participation, coping strategies, social support, and their collective influence on mental health. This exploration has underscored the nuanced differences between competitive and non-competitive athletes, revealing the intricate interplay between the type of sports involvement and its psychological outcomes. The findings illuminate the significant role of coping strategies and social support in mediating the relationship between sports participation and psychological well-being, offering a nuanced understanding that enriches the sports psychology field.

This study concludes that competitive and non-competitive sports participation distinctly impacts university students' psychological well-being. Competitive athletes rely on sophisticated coping mechanisms and a pronounced need for social support to navigate the pressures and challenges of their environment. In contrast, non-competitive athletes derive psychological benefits from their sports participation's intrinsic joy and leisure. These distinctions underscore the necessity of tailoring mental health interventions and support systems to the unique needs of different athlete groups, emphasizing the importance of a personalized approach in sports psychology and mental health care.

The theoretical implications of this research extend to enhancing our understanding of Self-Determination Theory, Stress and Coping Theory, Social Support Theory, and Competence Motivation Theory within the sports context. By integrating these psychological frameworks, the study validates their applicability and expands their reach, providing a deeper insight into the motivational and psychological processes underlying sports participation.

Several recommendations emerge from this study's findings for future research. Firstly, longitudinal studies are needed to track the psychological well-being of athletes over time, providing insights into the long-term effects of competitive and non-competitive sports participation. Such research could offer a more dynamic understanding of how coping strategies and social support evolve and interact with athletes' psychological well-being throughout their sports involvement.

Secondly, further exploration into the specific types of social support and coping strategies most effective for athletes in different sports contexts is warranted. This could involve qualitative studies that delve into athletes' experiences and perceptions, offering a richer, more detailed understanding of how sports participation impacts mental health.

Additionally, future research should consider the impact of emerging challenges, such as the global COVID-19 pandemic, on athletes' psychological well-being. Investigating how such crises affect athletes' mental health, coping strategies, and social support networks could provide valuable insights into resilience and adaptability.

Finally, there is an opportunity to explore the role of technology and digital platforms in supporting athletes' psychological well-being. With the increasing prevalence of online communities and digital mental health resources, examining their effectiveness in providing social support and facilitating coping among athletes could be a fruitful area of inquiry.

In conclusion, this study offers a comprehensive analysis of university students' psychological well-being in sports, highlighting the critical roles of coping strategies and social support. By providing a nuanced understanding of these dynamics, the research contributes valuable insights to the sports psychology field and offers practical guidance for educators, coaches, psychologists, and policymakers. Future research in this area promises to further our understanding of sports participation's psychological impacts, guiding the development of interventions and support systems that promote athletes' mental health and well-being across the spectrum of sports involvement.

## Funding statement

This article is supported by Basic scientific research business special project of central universities(ZY20240229).

## CRediT authorship contribution statement

**Jie Li:** Writing – review & editing, Writing – original draft. **Zhiyu Leng:** Visualization, Software, Data curation. **Kexin Tang:** Software, Formal analysis. **Meng Na:** Visualization, Investigation, Formal analysis. **Yixiang Li:** Supervision, Conceptualization. **Syed Shah Alam:** Supervision, Conceptualization.

## Declaration of competing interest

The authors declare the following financial interests/personal relationships which may be considered as potential competing interestsYixiang Li reports was provided by Xiangtan Institute of Technology. Yixiang Li reports a relationship with Xiangtan Institute of Technology that includes: employment. If there are other authors, they declare that they have no known competing financial interests or personal relationships that could have appeared to influence the work reported in this paper.
