# Supplementary material for: Multidimensional impact of sport types on the psychological well-being of student athletes: A multivariate investigation
Source: Heliyon. 2024 Jun 3;10(11):e32331. doi: 10.1016/j.heliyon.2024.e32331 (PMC11214501; doi:10.1016/j.heliyon.2024.e32331)
Supplement: Multimedia component 1 [file mmc1.doc]

**Multidimensional Impact of Sport Types on the Psychological Well-being of Student Athletes: A Multivariate Investigation**

**Dear Participant,**

Thank you for considering participation in our research study. The purpose of this investigation is to explore the multidimensional impact of different types of sports on the psychological well-being of student athletes. Your insights will contribute significantly to our understanding of how sporting activities influence mental health, coping strategies, emotional states, and overall happiness among student athletes.

This questionnaire is divided into several sections, each focusing on different aspects of your sports experience and its impact on your psychological well-being. The questions will cover your coping strategies, competitive and non-competitive sports involvement, emotional states, happiness, physical self-efficacy, satisfaction, social support, and the nature of your primary sport.

**Confidentiality and Anonymity**

Please be assured that your responses will remain confidential and anonymous. No personally identifiable information will be collected, and the data will be reported only in aggregate form. Participation is entirely voluntary, and you may withdraw at any time without penalty.

**Instructions**

For each statement, please indicate your level of agreement using the scale provided (1 = Strongly Disagree, 2 = Disagree, 3 = Neutral, 4 = Agree, 5 = Strongly Agree). Try to answer all questions honestly and to the best of your ability.

**Thank you for your valuable contribution to this study.**

**Demographics Information**

Please provide some basic information about yourself. This information will help us analyze the data more effectively and understand the diverse experiences of student athletes.

1. Please select the age range that applies to you.
   - 18-21
   - 22-25
   - 26-29
   - 30+
2. Please indicate your gender.
   - Male
   - Female
3. Please select your current year of study.
   - First Year
   - Second Year
   - Third Year
   - Fourth Year
4. Please select the category that best describes your major or field of study.
   - STEM (Science, Technology, Engineering, Math)
   - Humanities and Social Sciences
   - Business and Economics
   - Arts and Physical Education
5. Do you participate in sports competitively or non-competitively?
   - Competitive
   - Non-Competitive
6. Approximately how many hours per week do you dedicate to sports activities?
   - 0-2 hours
   - 3-5 hours
   - 6-10 hours
   - 11+ hours
7. Are you a member of any sports teams or clubs?
   - Yes
   - No

**Please indicate your level of agreement with the following statements:**

(1 = Strongly Disagree, 2 = Disagree, 3 = Neutral, 4 = Agree, 5 = Strongly Agree)

| **Coping Strategy** | | | | | | |
| --- | --- | --- | --- | --- | --- | --- |
| **COP1** | When faced with sports-related stress, I focus on what I can do to solve the problem. | 1 | 2 | 3 | 4 | 5 |
| **COP2** | I use relaxation techniques (e.g., deep breathing, meditation) to cope with stress in sports. | 1 | 2 | 3 | 4 | 5 |
| **COP3** | I seek advice or support from others when I encounter difficulties in my sports activities. | 1 | 2 | 3 | 4 | 5 |
| **Competitive Sprots** | | | | | | |
| **CS1** | Rate the level of competition in your primary sport (e.g., local, regional, national, international). | 1 | 2 | 3 | 4 | 5 |
| **CS2** | How often do you participate in competitive events or matches? | 1 | 2 | 3 | 4 | 5 |
| **CS3** | Do you engage in systematic training with the goal of improving competitive performance? | 1 | 2 | 3 | 4 | 5 |
| **Emotional States** | | | | | | |
| **ES1** | I generally feel positive about my life. | 1 | 2 | 3 | 4 | 5 |
| **ES2** | I feel emotionally balanced and stable. | 1 | 2 | 3 | 4 | 5 |
| **ES3** | I often experience feelings of joy and contentment. | 1 | 2 | 3 | 4 | 5 |
| **ES4** | I feel in control of my emotions, even in challenging situations. | 1 | 2 | 3 | 4 | 5 |
| **Happiness** | | | | | | |
| **HAP1** | Participating in sports activities makes me happy. | 1 | 2 | 3 | 4 | 5 |
| **HAP2** | I feel a sense of joy and excitement during or after sports activities. | 1 | 2 | 3 | 4 | 5 |
| **HAP3** | My sports experiences contribute positively to my overall happiness. | 1 | 2 | 3 | 4 | 5 |
| **Non-Competitive Sports** | | | | | | |
| **NCS1** | Do you participate in sports activities more for enjoyment than competition? | 1 | 2 | 3 | 4 | 5 |
| **NCS2** | How often do you engage in sports activities without keeping score or competing against others? | 1 | 2 | 3 | 4 | 5 |
| **NCS3** | Do your sports activities involve casual group participation or individual practice without a competitive goal? | 1 | 2 | 3 | 4 | 5 |
| **NCS4** | Do you engage in sports activities without a formal coach or training regimen? | 1 | 2 | 3 | 4 | 5 |
| **Physical Self Efficacy** | | | | | | |
| **PSE1** | I feel confident in my ability to meet the physical demands of my sport. | 1 | 2 | 3 | 4 | 5 |
| **PSE2** | I believe I can improve my sports performance through practice and effort. | 1 | 2 | 3 | 4 | 5 |
| **PSE3** | I am capable of learning new and challenging physical skills. | 1 | 2 | 3 | 4 | 5 |
| **Satisfaction** | | | | | | |
| **SAT1** | I am satisfied with my current level of sports performance. | 1 | 2 | 3 | 4 | 5 |
| **SAT2** | My participation in sports brings me a sense of fulfillment. | 1 | 2 | 3 | 4 | 5 |
| **SAT3** | I feel that my sports-related goals and desires are being met. | 1 | 2 | 3 | 4 | 5 |
| **SAT4** | I am content with the balance between my sports activities and other life areas. | 1 | 2 | 3 | 4 | 5 |
| **Social Support** | | | | | | |
| **SS1** | I feel supported by my teammates/coach/family in my sports endeavors. | 1 | 2 | 3 | 4 | 5 |
| **SS2** | My social network understands and respects the time I dedicate to sports. | 1 | 2 | 3 | 4 | 5 |
| **SS3** | I have someone in my sports circle I can talk to about problems related to sports. | 1 | 2 | 3 | 4 | 5 |
| **SS4** | When I am feeling down about my sports performance, I have people who uplift me. | 1 | 2 | 3 | 4 | 5 |
| **Sports Type** | | | | | | |
| **ST1** | My primary sport is team-based (e.g., football, basketball) / individual-based (e.g., tennis, swimming). | 1 | 2 | 3 | 4 | 5 |
| **ST2** | The nature of my primary sport is endurance-based (e.g., marathon, cycling) / strength-based (e.g., weightlifting). | 1 | 2 | 3 | 4 | 5 |
| **ST3** | My primary sport requires high physical contact (e.g., rugby, martial arts) / minimal physical contact (e.g., badminton, golf). | 1 | 2 | 3 | 4 | 5 |
| **ST4** | The environment of my primary sport is outdoor (e.g., soccer, rowing) / indoor (e.g., volleyball, gymnastics). | 1 | 2 | 3 | 4 | 5 |

We greatly appreciate your time and valuable insights in contributing to this research. Your participation is instrumental in advancing our understanding of the Impact of Sport Types on the Psychological Well-being of Student Athletes.
